# Attention capture by abrupt onsets: re-visiting the priority tag model

**DOI:** 10.3389/fpsyg.2013.00958

**Published:** 2013-12-20

**Authors:** Meera M. Sunny, Adrian von Mühlenen

**Affiliations:** ^1^Department of Psychology, Indian Institute of TechnologyGandhinagar, Ahmedabad, India; ^2^Department of Psychology, University of WarwickCoventry, UK

**Keywords:** FINST, attention, attention capture, abrupt onsets, priority tag

## Abstract

Abrupt onsets have been shown to strongly attract attention in a stimulus-driven, bottom-up manner. However, the precise mechanism that drives capture by onsets is still debated. According to the new object account, abrupt onsets capture attention because they signal the appearance of a new object. Yantis and Johnson (1990) used a visual search task and showed that up to four onsets can be automatically prioritized. However, in their study the number of onsets co-varied with the total number of items in the display, allowing for a possible confound between these two variables. In the present study, display size was fixed at eight items while the number of onsets was systematically varied between zero and eight. Experiment 1 showed a systematic increase in reactions times with increasing number of onsets. This increase was stronger when the target was an onset than when it was a no-onset item, a result that is best explained by a model according to which only one onset is automatically prioritized. Even when the onsets were marked in red (Experiment 2), nearly half of the participants continued to prioritize only one onset item. Only when onset and no-onset targets were blocked (Experiment 3), participants started to search selectively through the set of only the relevant target type. These results further support the finding that only one onset captures attention. Many bottom-up models of attention capture, like masking or saliency accounts, can efficiently explain this finding.

## Introduction

Attention capture by abrupt onsets is a robust and a fairly undisputed finding. It has been replicated many times using various methodologies (e.g., Todd and van Gelder, 1979; Yantis and Jonides, 1984; Theeuwes, 1991). One of the most commonly used methodologies is the *placeholder search paradigm* in which a preview display consisting of figure-8 placeholders is followed by a search display consisting of letters, along with a new letter at a previously un-occupied location (Yantis and Jonides, 1984). Participants search for a pre-specified target letter among various distractor letters. Typically Reaction Times (RTs) are faster when the target is the new item (the *onset item*) as compared to when it is one of the old items (the *no-onset items*). Also, RTs generally increase as a function of display size for no-onset targets, but not (or less so) for when the target is the onset item, suggesting that the abrupt onset is always processed first, irrespective of the number of items in the display. This effect occurs even when the type of target (onset or no-onset) is irrelevant to the search task (i.e., both onset and no-onset items are equally likely to be the target). Moreover, the capture effect seemed unique to abrupt onsets, as changes in other dimensions, like color or luminance, did not have the same effect on attention (Jonides and Yantis, 1988).

Nevertheless, there has been some debate about the mechanism underlying onset capture. According to Hillstrom and Yantis (1994), an abrupt onset constitutes the appearance of a new object in the visual field and, thus, instantiates the creation of an object file (cf. Kahneman et al., 1992), which requires the allocation of attention to the location of the object. They used letters that were perceptually new, but did not have an abrupt onset and showed that capture was mediated by the status of the letter as “new” rather than by their abrupt onset [also see Christ and Abrams (2006) for a similar finding using a slightly different method]. More evidence favoring the new object account comes from studies which show that illusory objects capture attention when these are perceived as new objects (Rauschenberger and Yantis, 2001; Yeshurun et al., 2009). However, the placeholder search paradigm remains the predominant methodology used to study onset capture.

Alternative accounts have been proposed to explain capture by abrupt onsets. For example, Miller (1989) argued that the capture effect comes from an increased local luminance change that occurs primarily at the location of the onset letter. That is, in a typical placeholder search paradigm, the local luminance change associated with the appearance of all the segments of the onset letter was usually larger than the selective disappearance of some segments of a no-onset letter. He showed that the capture effect disappeared when the overall change in luminance was held constant between the onset and the no-onset items^1^. Further support for this account comes from Watson and Humphreys (1995) who showed that increases and decreases in luminance (as occurs with onsets and offsets) had the same effect on attention when the overall change in luminance was kept constant. However, other studies have shown that a luminance change by its own cannot entirely account for the capture effect with abrupt onsets. For example, Enns et al. (2001) showed that a new object with a small luminance change was found faster than an old object with a large luminance change, supporting the new object account. Moreover, Gellatly et al. (1999) showed that a new object captures attention even when it was equiluminant with the background.

Yet another explanation of capture by abrupt onsets was offered by Gibson (1996a), who argued that an abrupt onset captures attention because it becomes available earlier than the no-onset items. In a series of experiments, he showed that search was faster in displays with all onsets as compared to displays with all no-onset items. He suggested that the placeholders preceding no-onset letters act as pre-masks, reducing their visual quality relative to that of onset items. This means, in the placeholder search paradigm the mask would slow down the processing of the no-onset item, and attention would simply be allocated to the first available item, the onset—the only item that would not be masked. Therefore, capture was put down to faster/better stimulus encoding of onsets rather than its status as a new object (but also see Gibson, 1996b; Yantis and Jonides, 1996). However, the masking account has also been criticized because no RT difference was observed between onset and no-onset stimuli in a detection task when attention was already allocated to the stimulus location (Yantis and Jonides, 1984; Yantis and Hillstrom, 1994). Thus, it remains an open question whether the onset effect results from an attentional advantage to the onset items or from a sensory deficit suffered by the no-onset items.

Further evidence for the special status of onsets in attention capture comes from the finding that up to four abrupt onsets are automatically prioritized in visual search (Yantis and Johnson, 1990; Yantis and Jones, 1991). Yantis and Johnson used a placeholder search paradigm with various display sizes (for example, 6, 8, 12, and 16 in Experiment 3), but with an equal number of onsets and no-onsets. Every trial started with the presentation of eight placeholders for 1 s. Then, depending on display size, placeholders disappeared (5, 4, 2, or 0, respectively) or changed to letters (3, 4, 6, or 8, respectively), and simultaneously, new letters appeared (3, 4, 6, or 8, respectively) such that the number of old (changed) objects and new (added) objects in the display were the same. The target was equally likely to be an onset or a no-onset item. They found that target type (onset or no-onset) interacted with display size between 6 and 8 items but not between 8 and 16 items. They took this as indirect evidence for their claim that participants selectively searched through up to four onsets before going through the remaining items in the display. They proposed a priority tag model, according to which all onsets are automatically priority tagged to be selectively searched. However, these tags decay over time and usually around four items are examined before the tags have decayed completely. Consistent with the idea that search times are directly linked to the rate of information extraction, Yantis and Jones (1991) showed that the number of items that was prioritized decreased from four to three when the visibility (i.e., visual quality) of the stimuli was reduced. Considering that onsets were not more likely to be the target than no-onset items, we would argue that the automatic prioritization of up to four items is somewhat surprising, given that observers had no incentive and no benefit from prioritizing that many items. That is, in spite of the target type being irrelevant to search, the onset set seems to get a large advantage during search, and consequently, the cost to the no-onsets would have been quite substantial.

This result seems to be in direct conflict with the salience based accounts of capture, which assume that capture effects are rather short lived and allows quick disengagement of attention (e.g., Kim and Cave, 1999; Donk and van Zoest, 2008). The theoretical implications of multi-element capture spans literature on attention capture in general as abrupt onsets are the only type of stimulus that survive the strictest test for capture using the irrelevant feature paradigm (Rauschenberger, 2003; Theeuwes, 2010). Hence it is important to establish the RT cost arising from having multiple onset distractors as well as to establish the RT advantage associated with the onset target.

In a series of three experiments we aim to see how the cost-benefit curve of onsets changes as a function of the number of onsets in the display. For a better estimate of the number of onsets that are automatically prioritized in visual search, it is suggested to keep the overall display size constant while the number of onsets is systematically varied. We believe this provides a better measure for attention capture than in Yantis and colleagues' study because an increase in RT is more directly linked to the number of onsets that capture attention, as there are no interference effects due to display size variations.

This would also give a more direct measure than Yantis and colleagues' paradigm as an increase in RT with an increase in the number of onsets would indicate that attention was captured by multiple onsets. An estimation of the number of items that capture attention can be done as described in the following section. The following model can fit a number of possible mechanisms, including the priority tagging model by which capture occurs. The focus in the present study is to provide an estimate of the number of onsets that are automatically prioritized and not to test a precise mechanism by which this is done. However, we will explain the models and make assumptions according to the priority tag model as this is the only model that currently accounts for simultaneous capture of multiple onsets.

## A formalized priority tag model

For simplicity, the priority tag model assumes that search operates as a serial self-terminating process^2^. Moreover, the model makes three assumptions: First, onsets are prioritized and there is a limit to the maximum number of items that can be prioritized. When the number of onsets exceeds the capacity of the priority set (i.e., there are more onsets than priority tags), a random subset of onsets is tagged. Second, search operates in two stages, where a subset of items (*the priority set)* is searched before the remaining items (*the non-priority set*). Third, within each subset, items are sampled in random order^3^.

This model determines *y*, which is the expected number of comparisons that are required to find the target. As it assumes a serial-self terminating search, the expected umber of comparisons are based on (*n* + 1)/2. However, the expected number of comparisons is also dependent on, first, the capacity of the priority set *c*, which refers to the maximum number of items that are tagged for priority processing. Second, it is dependent on three display factors: the total number of items (display size) *n*, the number of onsets *x*, and the target type *t*, that is, whether the target is an onset or a no-onset item^4^.

In the following equations, the calculation of *y* depends on two factors (yielding four equations): The first factor determines whether the priority set capacity is less than the number of onsets (*c* < *x*) or whether it exceeds or is equal to the number of onsets (*c* ≥ *x*). The second factor determines whether the target is an onset item or a no-onset item.

(1) $$\begin{array}{l} y(c;n,x)=\frac{c}{x}\cdot \frac{c+1}{2}+\left(1-\frac{c}{x}\right)\cdot \left(c+\frac{n-c+1}{2}\right),\\ \text{ for }c<x,t=\text{onset} \end{array}$$

(1a) $$y(c;n,x)=\frac{n+c+1}{2}-\frac{cn}{2x},\text{for }c<x,t=\text{onset}$$

Equation 1 gives the expected number of comparisons *y* required to find the target when it is an onset (*t* = onset) and when the priority set capacity is smaller than the number of onsets (*c* < *x*). Because there are more onsets than priority tags, a random subset of onsets (the priority set) is tagged and searched. This first stage is represented in the first expression in the right of equation 1, which multiplies the probability of the target being in the priority set (*c*/*x*) by the expected number of comparisons required to find the target in that set (*c* + 1)/2. When the target is not in the priority set, search continues to scan through the remaining items (the non-priority set) in random order, irrespective of whether these are onset or no-onset items. This second stage is represented in the second expression of Equation 1, where the probability of the target being in the non-priority set (1 − *c*/*x*) is multiplied by the expected number of comparisons required to find the target when it is in that set. This latter number comprises searching the entire priority set (*c*) and finding the target in the non-priority set (*n* − *c* + 1)/2. Equation 1 can be simplified to Equation 1a.

(2) $$y(c;n,x)=\frac{x+1}{2},\text{for }c\geq x,t=\text{onset}$$

Equation 2 gives *y* for when the target is an onset (*t* = onset) and when the priority set capacity is greater than or equal to the number of onsets (*c* = *x*). Because there are fewer onsets than priority tags, all onsets are tagged, and because the target is an onset only the priority set needs to be searched. Hence, the expected number of comparisons required to find the target depends only on the number of onsets (*x*).

(3) $$y(c;n,x)=c+\frac{n-c+1}{2},\text{for }c<x,t=\text{no-onset}$$

(3a) $$\;y(c;n,x)=\frac{n+c+1}{2},\text{for }c<x,t=\text{no-onset}$$

Equation 3 gives *y* for when the target is a no-onset (*t* = no-onset) and the priority set is smaller than the number of onsets (*c* < *x*). Because there are more onsets than priority tags, a random subset of onsets is tagged. Moreover, because the target is a no-onset item, the entire priority set (of onsets) must be searched first, what is represented by *c* in the first expression of Equation 3. The second expression of the equation shows the expected number of comparisons required to find the target in the non-priority set (*n* − *c* + 1)/2. Equation 3 can be simplified to Equation 3a.

(4) $$y(c;n,x)=x+\frac{n-x+1}{2},\text{for }c\geq x,t=\text{no-onset}$$

(4a) $$y(c;n,x)=\frac{x+n+1}{2},\text{for }c\geq x,t=\text{no-onset}$$

Finally, Equation 4 gives *y* for when the target is a no-onset item (*t* = no-onset) and the priority set capacity is greater than or is equal to the number of onsets (*c* ≥ *x*). Because there are fewer or equal numbers of onsets than priority tags, all onsets are tagged, and because the target is a no-onset the entire priority set (of onsets) needs to be searched first, what is represented by *x* in the first expression of Equation 4. The second expression of the equation represents the expected number of comparisons required to find the target in the non-priority set (*n* − *x* + 1)/2. Equation 4 can be simplified to Equation 4a.

**Figure 1:**
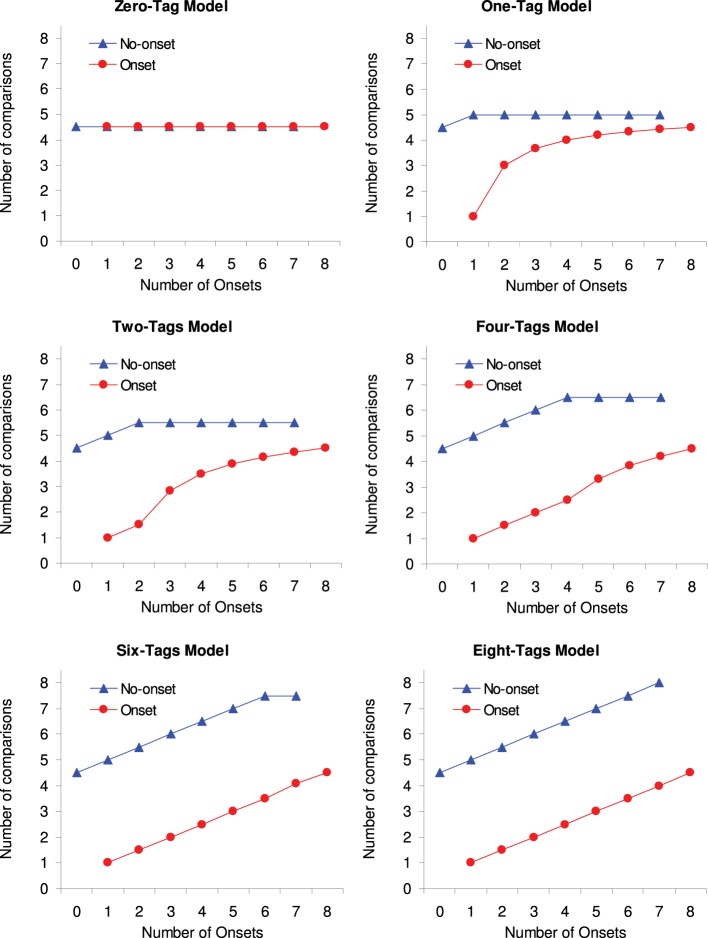
**Expected number of comparisons *y* as a function of number of onsets *x* with separate lines for onset and no-onset targets.** Each graph represents a different capacity *c*.

Figure 1 shows the expected number of comparisons for a fixed display size of eight items (*n* = 8). Each graph shows the expected number of comparisons for a given capacity (*c* = 0, 1, 2, 4, 6, and 8) as a function of number of onsets (*x* = 0 to 8), with separate lines for when the target is an onset or a no-onset item. As can be seen, when no onset is tagged (*c* = 0) then the number of onsets has no effect on RT, whereas when all onsets are tagged, (*c* = 8) then RTs increase linearly with number of onsets, at the same rate for onsets and no-onset targets (even though onset targets require on average four comparisons less than no-onset targets). For all intermediate levels of *c* the model predicts some form of interaction between the target type and the number of onsets.

In order to estimate the best fitting value of *y* in the proposed model above, behavioral data was collected and fitted against the different model predictions. In a first experiment, the display size was fixed at eight while target type (onset, no-onset) and number of onsets (*x* = 0, 1, 2, 4, 6, or 8) were systematically varied. The aim of the experiment was to determine the value of *c* that provides the best fit between the model and the data. This value would determine the maximum number of onsets that would be processed automatically with priority.

## Experiment 1

Experiment 1 used a placeholder search paradigm where the number of onsets was systematically varied between zero and eight. The display size was fixed at eight items so that changes to the number of onsets was the only factor determining changes in attentional prioritization, avoiding possible confounds between the number of onsets and the display size.

### Methods

#### Participants

In this and in all subsequent experiments, students from the University of Warwick participated in return for course credit. They all reported normal or corrected to normal visual acuity, they were naïve to the purpose of the experiment, and they participated only once. The present experiment had 22 participants (8 males, 14 females, mean age 19.4 years).

#### Apparatus and stimuli

**Figure 2:**
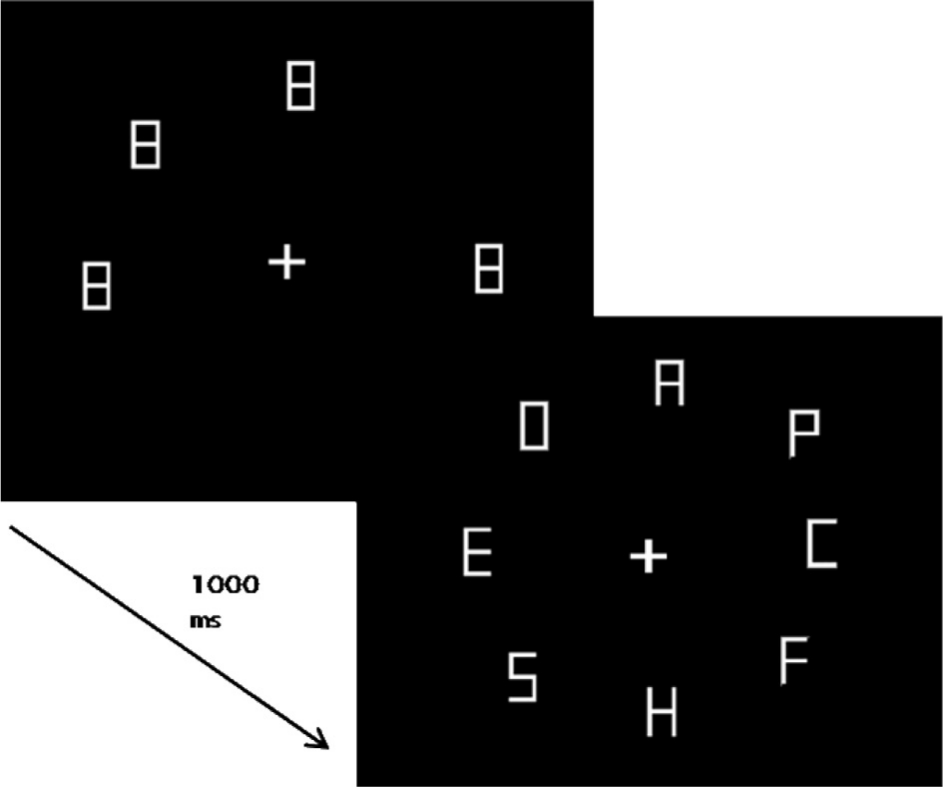
**An example display showing the sequence of events in Experiment 1 with four onsets and four no-onset items (preceded by figure-8 placeholders).** Display size was fixed at 8, but the number of onsets varied systematically from 0, 1, 2, 4, 6, to 8.

The participants were seated in a dimly lit sound attenuated room in front of a 19″ CRT monitor at a distance of approximately 57 cm. The monitor was driven at 100 Hz at a resolution of 1024 × 786 pixels. The experiment was controlled by an IBM-PC compatible computer using custom written software. Participants' responses were recorded using the left and right arrow keys on a standard keyboard. Stimuli consisted of a fixation cross, figure-8 placeholders, and letters, presented in gray (luminance 8.5 cd/m2) drawn on black background (0.02 cd/m2). The fixation-cross subtended a visual angle of 0.6 × 0.6° and was presented at the center of the screen. The figure-8 placeholders and letters subtended 1 × 2° and were made of seven line segments (length 1.0°, thickness 0.13°). The letters “H” and “U” served as targets and, “S,” “E,” “F,” “O,” “C,” “P,” and “A” as distractors. The letters were made by removing the corresponding line segments from the figure-8. The stimuli were placed on the circumference of an imaginary circle (radius 12.5°) centered on fixation, such that the letters were equidistant from each other (see Figure 2).

#### Procedure and design

A trial started with the presentation of a preview display that consisted of a fixation cross and figure-8 placeholders presented for 1000 ms (see Figure 2). The number of placeholders was systematically varied between 8, 7, 6, 4, 2, and 0. The preview display was followed by the search display, which always contained eight letters. Hence, depending on the number of placeholders, the search display had 0, 1, 2, 4, 6, or 8 onset items, respectively. The no-onset letters were revealed by deleting the irrelevant line segments from the corresponding placeholders, whereas the onset letters always appeared at previously unoccupied locations (see Figure 2). The target was equally likely to be an onset or a no-onset.

Participants were asked to search for the target and to respond with the left or right arrow keys. Half of participants pressed the left key for “H” and the right key for “U,” and vice versa for the other half. They were instructed to respond to the target as quickly as possible whilst trying not to make more than 5% errors. They were also told that the preceding placeholders were not task-relevant and that the target would appear with equal likelihood at any of the eight possible locations. The search display stayed on until the participant responded or 10 s had elapsed. Trials with no response within 10 s were marked as an error. In the instance of wrong responses, immediate feedback was displayed on the screen saying “error” and participants had to press the space bar to continue with the next trial. The inter-trial interval was 1 s. The proportion of trials where the target was an onset was inversely related to the number of onsets in the display, in order to ensure that the target was not more likely to be an onset than a no-onset item. For example, when the display contained one onset, the target was the onset in 1/8 of the trials and a no-onset in the remaining 7/8 of the trials, but when the display contained four onsets, the target was an onset in half the trials or a no-onset in the other half of trials (the number of trials in each condition is also given in Table 1).

**Table 1:** **Number of trials (*N*) and percentage errors (%) for different target type and number of onsets in Experiment 1**.

**Number of onsets**	**Target type**			
	**No-Onset**		**Onset**	
	***N***	**Error (%)**	***N***	**Error (%)**
0	20	4.1	0	−
1	140	4.3	20	2.4
2	60	4.6	20	2.2
4	20	4.8	20	2.8
6	20	4.3	60	3.0
8	0	−	20	3.9
Total (Mean)	260	(4.4)	140	(2.9)

Each participant completed 20 practice trials followed by 400 experimental trials. The experimental trials were divided into eight blocks of 50 trials each, with short enforced breaks between blocks. The experiment systematically varied three factors: target identity (H or U), target type (no-onset, onset), and number of onsets (0, 1, 2, 4, 6, or 8). All possible factor combinations were presented in random order. In the analysis, target identity was not considered.

### Results

#### RTs

**Figure 3:**
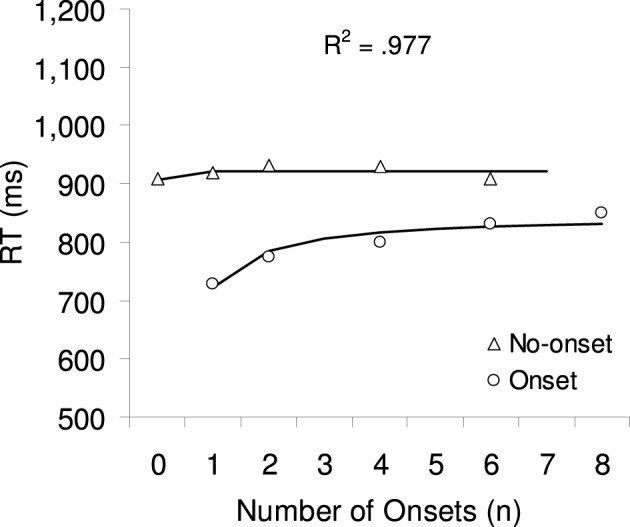
**The symbols show the mean correct RTs for onset and no-onset targets in Experiment 1.** The lines and the R2 show the result of a multiple regression analysis, predicting RT based on the 1-tag model, which provided the best fit.

Mean correct RTs were calculated separately for each participant and factor combination, excluding outlier trials with RTs smaller than 200 ms or larger than 2000 ms (1.1% of all trials). Figure 3 shows the averaged RTs as a function of number of onsets with separate lines for each target type. A 2 × 4 Repeated Measures ANOVA with the factors target type (no-onset or onset) and number of onsets (1, 2, 4, or 6, excluding 0 and 8 onsets in order to have a fully factorial design) was calculated. Greenhouse-Geisser corrections were used in this and all subsequent ANOVAs. There was a significant main effect of target type *F*_(1, 21)_ = 96.51, *p* < 0.001: onset targets were found on average 113 ms faster than no-onset targets. The main effect of number of onsets was also significant *F*_(3, 63)_ = 6.79, *p* < 0.005: RTs increased in total by 46 ms from one onset to six onsets. Moreover, there was a significant 2-way interaction, *F*_(3, 63)_ = 8.23, *p* < 0.001, which was further explored with two separate 1-way ANOVAs with the factor number of onsets (including 0 and 8 onsets where available). There was no significant effect when the target was a no-onset item, *F* < 1, but a highly significant effect when the target was an onset, *F*_(4, 84)_ = 16.18, *p* < 0.001: RTs increased on average by 103 ms from one onset to six onsets.

#### Errors

Mean percentage errors were calculated separately for each participant and factor combination (see Table 1). Error rates were relatively low (on average 3.7%), suggesting that participants had no problems following the instruction to keep the error rate below 5%. A 2 × 4 repeated measures ANOVA with the factors target type (no-onset, onset) and number of onsets (1, 2, 4, or 6 items) revealed a significant main effect for target type, *F*_(1, 21)_ = 6.03, *p* < 0.05, due to the somewhat higher error rate for no-onset than for onset targets (4.4 vs. 2.9%, respectively). Although the other effects were not significant (both *F* < 1), errors showed overall a very similar pattern to the RTs, suggesting that RTs were not confounded by speed-accuracy trade-offs.

#### Model fitting

Eight multiple regressions were calculated to determine the model parameter ***c*** that provided the best fit for the RTs observed in Experiment 1. The expected number of comparisons, *y* (as predicted by Equations 1–4), are taken as a direct estimate for the RT (see also Figure 1). Previous studies have shown that RTs are generally faster when a search display contains only onset as compared to when it contains only no-onset items (e.g., Gibson, 1996a). However, our model does not account for such a difference, as can be seen from Figure 1, where the expected number of comparison in these two cases is always 4.5, irrespective of the priority set capacity. In order to allow a better fit of our data, we have incorporated this difference by adding the predictor target type *t*(0, 1) to the regression, which will add the weight *b* to the RT when the target is an onset (*t* = 1).

(5) RT(c)=a·y(c;n,x)+b·t+d

The regression weight (*a*) represents the search rate (ms/item) as it stands for the expected number of comparisons as predicted by the *c*- tag model (see Equations 1–4); the regression weight (*b*) represents the onset advantage as it stands for target type (0 = onset, 1 = no-onset); finally the constant (*d*) represents the time taken for all other processes involved in making the visuo-motor response. The results of the regressions analyses are presented in Table 2, separately for each value of number of priority tags (*c* = 1, 2, 3, 4, 5, 6, 7, or 8). As can be seen in the last column, the 1-tag model provides the best fit (*r*^2^ = 0.977), with the goodness of fit continuously decreasing as the number of tags increase.

**Table 2:** **Summary of eight separate regression analyses predicting RT in Experiment 1 for the various priority tag models**.

***C***	***a* (*SE*)**	***b* (*SE*)**	***d* (*SE*)**	***R*^2^**
0	−^a^	−^a^	−^a^	−^a^
1	31.6 (4.2)	−74.1 (10.3)	764 (21.7)	0.977
2	27.7 (4.0)	−59.5 (12.4)	774 (21.8)	0.974
3	26.7 (4.7)	−52.9 (16.0)	774 (26.8)	0.963
4	23.6 (5.8)	−53.1 (21.3)	786 (33.8)	0.939
5	21.4 (6.4)	−56.8 (24.6)	797 (38.3)	0.920
6/7/8^b^	18.8 (6.9)	−62.4 (27.5)	810 (41.7)	0.900

a The 0-priority tag model predicts no variation.

b The 6-, 7-, and 8-priority-tag models make the same prediction for this data set.

In order to allow a statistical test comparing the goodness of fit for the various tag-models, the regressions were calculated individually for each participant and each number of priority tags (*c*). A one-way ANOVA on these resulting *R*^2^ values with the factor number of priority tags showed a significant effect, *F*_(5, 105)_ = 6.78, *p* < 0.01. *Post-hoc* LSD comparisons revealed that the difference between each level pair was significant (all *p* < 0.05), except the difference between the 1-tag model and the 2-tag model (*p* = 0.34). In other words, the 1–2 tag models provide a significantly better fit than the 3–8 tag models. Also, the 3-tag model provides a better fit than the 4–8 tag models, the 4-tag model than the 5–8 tag models, and the 5-tag than the 6–8 tag models.

### Discussion

In Experiment 1, we tested the effect of simultaneous multiple onsets on attention capture. We used search displays with eight items, while systematically varying the number of onsets. The results showed a RT pattern that was most similar to the pattern of expected number of comparisons as predicted by the 1-tag model (cf. Figures 1, 2). For example, the finding that RTs for onset targets increased most between one onset and two onsets was predicted only by the 1-tag model. Also, the fact that RTs did not change much for no-onset targets also fits best with the 1-tag model. This interpretation was further confirmed by multiple regression analyses, which showed the best fit for the 1-tag model. The fit for the 1-tag model was significantly better than the fit for all the other models, except for the 2-tag model. However, even though the data is equally well fitted by the 2-tag and the 1-tag model, we would argue that the 1-tag model provides a better explanation of the data, as it won't rely on a tagging mechanism, in order to explain onset capture. Other simpler accounts (e.g., Gibson, 1996a, or Donk and van Zoest, 2008) could explain capture by onsets in the absence of capture by multiple onsets.

The present findings, however, are in stark contrast to previous studies showing that up to four onsets are automatically prioritized in search (Yantis and Johnson, 1990; Yantis and Jones, 1991). While we fixed display size at eight and varied only number of onsets, Yantis and colleagues varied both display size (4–16 items) and number of onsets (2–8 onsets) together. One possible explanation for the difference between their and our study could be that in their study the deflection point in the RT slope was skewed by a commonly observed flattening of the RT slope at the larger display sizes (e.g., Wolfe et al., 2010). In other words, the gradual flattening of the RT slope in Yantis and Johnson (1990) third experiment might have been wrongly interpreted as a deflection point occurring at display size eight (as was predicted by the four-tag model).

The present results can be better explained by a purely bottom-up account of attention capture. Searching for a letter among other letters is only a moderately difficult task, and we would expect that after the initial capture, search becomes more guided by the target identity than by the target type, as the latter is entirely task irrelevant. Hence we would expect that only one onset (if present) is inspected with priority. This corresponds to a purely bottom up model of attention capture, which suggest that capture is triggered by an increased saliency signal that accompanies abrupt display changes (Theeuwes, 2010). Recent studies have shown that such salience signals can be very short-lived (e.g., Donk and van Zoest, 2008). Re-entrant processes take over after the initial feed forward sweep, and the identity of the letters could be actively prioritized over its onset status (Di Lollo et al., 2000). Capture by a single onset fits well with models according to which the initial boost enjoyed by abrupt onsets is not sustained beyond the inspection of the first element. The present experiment therefore suggests that attention capture is better explained by a bottom-up, salience based model than an automatic priority tagging model.

## Experiment 2

Experiment 1 suggests that, in a display with multiple simultaneous onsets, only one onset is automatically prioritized. This result fits well with bottom-up theories of capture where the onset advantage is short lived and search quickly turns to object identity rather than onset status in order to find the target. One possible reason for a short-lived onset advantage might be that it was not possible to distinguish between onset and no-onset items once the letters were revealed. Maybe capture by multiple onsets would be facilitated if the onsets were in some way distinguishable from the other items for the duration of the search. In Experiment 2, the onset items were therefore presented in red, distinguishing them from the no-onset items (which remained gray). This distinctive feature marked the onsets throughout the entire search process, preventing the decay of the onset information. In Experiment 2 it was thus expected that the color difference would help to tag and prioritize more than one onset item. Moreover, in Experiment 2, two additional levels for number of onsets (3 and 5 onsets) were included in order to allow for better fit of the various tag models.

### Methods

Twelve participants (3 male, 9 female, mean age 19.5 years) took part in this experiment. The apparatus, stimuli and procedure were the same as in Experiment 1, except that all onset letters were presented in red (luminance 6.4 cd/m2). The design was similar to that of Experiment 1, except that number of onsets had two additional levels (3 and 5 onsets), increasing the total number of trials to 560 (see also Table 3).

**Table 3:** **Number of trials (*N*) and mean percentage errors (%) for different target type and number of onsets in Experiment 2**.

**Number of onsets**	**Target type**			
	**No-onset**		**Onset**	
	***N***	**Error (%)**	***N***	**Error (%)**
0	20	2.5	0	−
1	140	3.5	20	2.1
2	60	4.6	20	1.4
3	50	4.4	30	3.3
4	20	3.6	20	1.8
5	30	3.6	50	3.9
6	20	3.9	60	4.2
8	0	−	20	2.1
Total (Mean)	340	(3.7)	220	(2.7)

### Results

#### RTs

**Figure 4:**
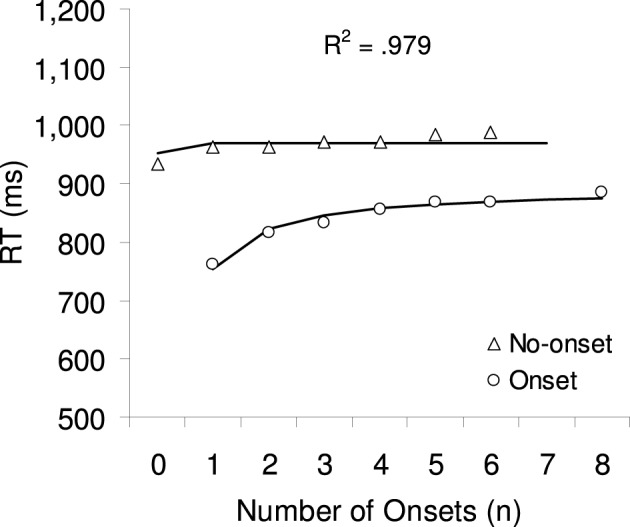
**The symbols show the mean correct RTs for no-onset and onset targets in Experiment 2.** The lines and R2 show the best fit from a multiple regression analysis, predicting RT based on the 1-tag model, which provided the best fit.

Mean correct RTs were calculated separately for each participant and factor combination, excluding 1.9% outlier trials (see Figure 4). A 2 × 6 ANOVA with the factors target type (no-onset or onset) and number of onsets (1, 2, 3, 4, 5, or 6, excluding 0 and 8 to have a fully factorial design) showed a significant main effect of target type *F*_(1, 11)_ = 24.44, *p* < 0.001: onset targets were found on average 139 ms faster than no-onset targets. The main effect of the number of onsets was also significant *F*_(5, 55)_ = 3.68, *p* < 0.05: RTs increased on average by 25 ms from one onset to six onsets. The two-way interaction between target type and number of onsets did not reach significance *F*_(5, 55)_ = 1.39, *p* = 0.27. Two separate 1-way ANOVAs were calculated with the factor number of onsets, in order to include the level 0 onset (no-onset target) and the level 8 onsets (onset-target). There was no significant effect when the target was a no-onset item, *F* < 1, but a highly significant effect when the target was an onset, *F*_(6, 66)_ = 4.99, *p* < 0.005, due to an RT increase of 122 ms from 1–8 onsets.

#### Errors

Mean percentage errors were calculated separately for each participant and factor combination (see Table 3). Overall error rates were relatively low (on average 3.2%). A 2 × 6 repeated measures ANOVA with the factors target type (no-onset, onset) and number of onsets (1, 2, 3, 4, 5, or 6 items) revealed a marginally significant main effect for target type, *F*_(1, 11)_ = 4.78, *p* = 0.051, due to the somewhat higher error rate with no-onset targets than with onset targets (3.7 vs. 2.7%, respectively). Again, there was no indication that RTs were confounded by speed-accuracy trade-offs.

#### Model fitting

As in Experiment 1, eight multiple regression analyses using Equation 5 were used to determine the model parameter *c* that provides the best fit for the RTs observed in Experiment 1. As can be seen from the last column in Table 4, again, the 1-tag model and 2-tag model provide the best fit (*R*^2^ = 0.979 and 0.980, respectively), with the goodness of fit continuously decreasing as the number of tags increase. Note that although the values providing the best fits were of similar magnitude, the *R*^2^ range was much smaller in this experiment (0.955–0.980) than in the previous experiment (0.900–0.977). This observation was also confirmed in a one-way ANOVA on the individual *R*^2^, which failed to show a significant effect for number of priority tags, *F* < 1. Thus, despite this range of *R*^2^ values, statistically, all models provide an equally good fit for the RT data in Experiment 2.

**Table 4:** **Summary of eight separate regression analyses predicting RT in Experiment 2 for the various models**.

***C***	***a* (*SE*)**	***b* (*SE*)**	***c* (*SE*)**	***R*^2^**
0	−^a^	−^a^	−^a^	−^a^
1	34.7 (3.8)	−77.9 (8.0)	797 (19.0)	0.979
2	30.5 (3.2)	−58.4 (9.4)	806 (17.7)	0.980
3	29.1 (3.4)	−45.8 (11.4)	805 (19.5)	0.976
4	27.2 (3.9)	−41.2 (14.4)	810 (23.2)	0.966
5	25.6 (4.1)	−41.1 (16.1)	816 (25.2)	0.959
6/7/8^b^	24.4 (4.2)	−42.8 (17.0)	821 (26.1)	0.955

a The 0-priority tag models predicts no variation.

b The 6-, 7-, and 8-priority-tag models make all the same prediction.

### Discussion

Experiment 2 tested whether adding a distinctive feature to all onsets changed their attentional priority. As in Experiment 1, the best fit seemed to come from a model where only one or two onsets capture attention. A comparison of the RT pattern in Figure 3 with the model predictions in Figure 1, suggests that the RT data is best predicted by a model in which only one onset captures attention. For example, the RT increase for onset targets was again at its peak between one and two onsets; and RTs did not change much for no-onset targets, except from zero onset to one onset (*p* = 0.07), corresponding to the predictions of the 1-tag model. However, in comparison to Experiment 1, these differences between the 1–2-tag and the 2–8-tag models did not reach statistical significance. A possible explanation for this smaller range of *R*^2^ could be based on individual differences in the distribution of best-fit *R*^2^ values across participants. In Experiment 1, the data from the majority of the participants (18 out of 22) seemed to fit with a 1–2-tag model, whereas in Experiment 2 only half of the participants' data (6 out of 12) seemed to fit with a 1–2-tag model. This indicates that when the onsets were red, more participants were using a multi-tag strategy, where they paid particular attention to the colored onset items. This can be taken as preliminary evidence that the color difference might have affected at least some of the participants.

Nevertheless, it is important to note that at least half of the participants appeared to have used a 1–2-tag model. This suggests that increasing the saliency of the onsets by coloring those red did not change the capture effect in these participants. Because, the red color (like target type) did not predict the target location, participants had no incentive to attend more to the onset than to the no-onset items. That is, it seems that after an initial reflexive capture of attention by one or two abrupt onset, participants were subsequently able to overcome capture and to exert top-down control in order to focus on the task at hand. The finding that automatic capture is limited to one or two onsets (at least in half the participants), is again not in line with the findings of Yantis and colleagues (Yantis and Johnson, 1990; Yantis and Jones, 1991) who reported that up to four onsets can capture attention. The next experiment will further explore the limits for automatic attentional allocation to more than one item.

## Experiment 3

The results of Experiment 1 suggest that the majority of participants (82%) automatically prioritized and searched one or two abrupt onset. The final experiment investigates whether it is possible to prioritize multiple onsets when participants knew in advance what the target type was going to be. Moreover, it also tests whether the same prioritizing occurs with no-onset items (when it is known that the target is amongst them). Top-down prioritization by onsets was tested by presenting the factor target type in separate blocks so that in each block participants knew whether the target was an onset or a no-onset item. This means adopting a top-down set for selectively processing either onsets or no-onsets in different blocks of the experiment. Being able to tag multiple onsets (or no-onsets) becomes now beneficial to the task, as it allows prioritizing potential target letters. Although selectively searching through no-onsets could be beneficial to the task, participants would first have to overcome the processing advantage that is typically associated with abrupt onsets.

For modeling purpose, this means that the first assumption needs to be changed, such that both target types (not only onset) can be tagged. That is, either onset or no-onset items are automatically priority tagged, depending on which target type is (in the current block) relevant for the task; when the number of search relevant items exceeds the number of priority tags, a random subset of them is tagged. However, we can still operate by the second assumption (division into priority and non-priority set) and the third assumption (random order within each subset).

### Methods

Twelve participants (3 males, 9 females, mean age 18.5 years) participated in this experiment. The apparatus, stimuli, procedure and design were the same as in Experiment 1, with the following differences: Target type (onset, no-onset) was blocked and presented in two consecutive sessions. Half the participants started with onset followed by no-onset targets, and vice-versa for the other half. At the beginning of each session participants were told that the target was either always an onset item (with no placeholder) or always a no-onset item (with placeholder). The number of onsets was systematically varied from 0, 1, 2, 3, 4, 5, 6, 7, to 8 onsets, with a fixed number 32 trials for each combination of target type and number of onsets. Thus, the experiment had a total of 512 trials (see also Table 5).

**Table 5:** **Number of trials (*N*) and mean percentage errors (%) for different target type and number of search-relevant items in Experiment 3**.

**Number of search-relevant items**	**Target type**			
	**No-Onset**		**Onset**	
	***N***	**Error (%)**	***N***	**Error (%)**
1	32	4.2	32	2.9
2	32	2.6	32	6.0
3	32	3.4	32	2.6
4	32	4.2	32	3.9
5	32	4.7	32	2.3
6	32	5.2	32	4.2
7	32	4.7	32	3.4
8	32	2.1	32	4.7
Total (Mean)	256	(3.9)	256	(3.8)

### Results

#### RTs

**Figure 5:**
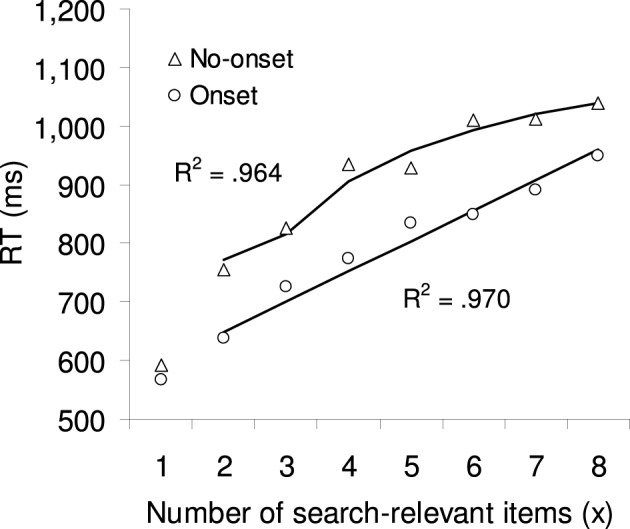
**The symbols show the mean correct RTs for no-onset and onset targets in Experiment 3.** The lines and R2 show the best fit from a multiple regression analysis (excluding the level with one search relevant item), predicting RT based on the 3-tag model.

Mean correct RTs were calculated separately for each target type and number of onsets combination, excluding 0.9% outlier trials. In order to allow better comparison between onset and no-onset target types, Figure 5 plots RTs as a function of number of search relevant items (instead of number of onsets). A 2 × 8 ANOVA with the factors target type (no-onset or onset) and number of search-relevant items (1, 2, 3, 4, 5, 6, 7, or 8) showed a significant interaction, *F*_(7, 77)_ = 3.61, *p* < 0.05. This interaction was due to the absence of a target type effect when the number of search relevant items was one, *t*_(11)_ = 1.91, *p* = 0.083. We assume that this was because the target location was always 100% accurately predicted when the search relevant item was one, either by the one placeholder that was present in the preview display or by the one placeholder that was missing. We therefore calculated a separate 2 × 7 ANOVA which was excluding this one level. This ANOVA showed a significant main effect of target type *F*_(1, 11)_ = 32.09, *p* < 0.001: Onset targets were found on average 108 ms faster than no-onset targets. And it showed a significant main effect of number of search-relevant items *F*_(6, 66)_ = 54.70, *p* < 0.001: RTs increased on average by 298 ms from 2–8 search-relevant items. However, there was no significant interaction, *F*_(6, 66)_ = 1.61, *p* = 0.20, indicating that the two main effects were independent of each other^5^.

#### Errors

Mean percentage errors were calculated separately for each participant and factor combination (see Table 5). Overall error rates were relatively low (on average 3.8%). A 3 × 7 repeated measures ANOVA with the factors target type (no-onset, onset) and number of search-relevant items (2–7) revealed no significant effects (all *p* > 0.17). Again there was no indication that RTs were confounded by speed-accuracy trade-offs.

#### Model fitting

Similar to Experiments 1 and 2, regression analyses were used to determine the model parameters that made the best predictions for the RTs observed in Experiment 3. Participant always knew the relevant target type because of the blocked design. To account for this knowledge, we applied Equations 1 and 2 (which used to be for onset target only) to both the onset and the no-onset conditions. Furthermore, because of the blocked design, separate regressions were calculated for onset- and for no-onset targets. This also accounts for any difference in the strategies that the participants might have adapted for the different target type conditions. The new regression in Equation 6 had therefore only one factor (*a*), representing the search rate.

(6) RT(c)=a·y(c;n,x)+d

The results of eight regression analyses calculated separately for no-onset targets and each number of priority tags (*c*) are given in the top half of Table 6, and the corresponding regression analyses for onset targets are given in the bottom half of Table 6. Looking at the *R*^2^ in the last column, it can be seen that the 3-tag model provides the best fit for no-onset targets, whereas the 8-tag model provides the best fit for onset targets. This was also confirmed in two separate one-way ANOVA on the individual *R*^2^, which both showed a significant effect for number of task-relevant items (both *p* < 0.01).

**Table 6:** **Summary of 16 regression analyses predicting RT in Experiment 3 separately for onset and no-onset targets for various priority tag models**.

***C***	***a* (*SE*)**	***d* (*SE*)**	***R*^2^**
**NO-ONSET**			
1	191.7 (21.3)	158 (86.6)	0.941
2	95.8 (10.6)	589 (39.2)	0.942
3	89.2 (7.7)	637 (26.5)	0.964
4	87.6 (11.7)	655 (38.6)	0.918
5	89.9 (12.1)	654 (38.9)	0.917
6	91.5 (13.1)	653 (41.7)	0.906
7/8^a^	92.6 (13.1)	650 (41.4)	0.909
**ONSET**			
1	190 (23.2)	42 (93.9)	0.931
2	95 (11.6)	471 (42.5)	0.931
3	88 (8.4)	517 (29.0)	0.957
4	89 (8.1)	528 (26.8)	0.960
5	91 (8.6)	528 (27.8)	0.957
6	94 (7.8)	523 (24.9)	0.967
7/8^a^	95 (7.5)	521 (23.9)	0.970

a The 7- and 8-priority-tag models make the same prediction.

### Discussion

Experiment 3 tested whether participants can prioritize a subset of items when they had prior knowledge of the target type. The results showed that when the target was an onset, RTs linearly increased with each additional onset, indicating that participants were able to priority tag multiple—possibly even all—onset items. The results showed a similar pattern for when the target was a no-onset item, with a similar increase in RTs with increasing number of no-onset items. However, the regression analyses suggested that participants were able to priority tag only up to three no-onset items. Moreover, RTs for onset and no-onset targets were the same when only one of its types was present in the display.

The RTs for the onset items were overall 120 ms faster as compared to the RTs for the no-onset items. This suggests that to an extent, an onset obtained some processing advantage, leading to an RT benefit for the onset target and cost to a no-onset target. The results, however do not dissociate between these two effects. One possible way by which multiple onsets are prioritized during search can be explained by theories of visual marking. In visual marking, participants actively inhibit preview location in order to selectively process the relevant set of distractors (Watson and Humphreys, 1997). In the present study, participants could thus inhibit the object locations in which the placeholders appear, resulting in selective processing of the new items. Note that other studies have previously suggested that the preview effect described above was not due to the inhibition of old stimuli already in the field, but due to the fact that new items capture attention automatically simply because they generate luminance onset signals (e.g., Donk and Theeuwes, 2001, 2003).

The inability to select more than 3 no-onsets suggests that a different mechanism might work in selectively searching through the no-onset items. A possible explanation for this difficulty in selecting multiple items might come from the visual indexing theory—also known as FINST [for “fingers of instantiation,” Pylyshyn (1989)]. According to FINST, it is possible to index or tag a small numbers of items in the visual field and these indexes can be used to track changes to these object. This indexing mechanism is controlled in a top-down manner and is limited to about 4 items. The present results with no-onset targets are more or less consistent with this prediction of FINST. Moreover, it is assumed that this indexing is object-based, making it impossible to index empty locations. So, this visual indexing would not work in the same way when searching for an onset target. One possibility could be that selective search through onsets proceed by inhibiting all the no-onset items, whereas search through the no-onsets proceed by indexing a subset of the no-onsets and tracking them.

The results of Experiment 3 also show that the overall onset advantage discussed above was absent when there was only one search-relevant item. In these conditions, given the specific design of the experiment, the target position was always known in advance (i.e., the target location was indicated by the presence or absence of one placeholder). In this sense the placeholder or empty location acted like a spatial cue that was always valid. It is interesting that in this condition, both onset and no-onset targets were detected with equal speed, thus providing another case where an onset item had no advantage over a no-onset item. This is in line with findings that under focused attention conditions, abrupt onsets do not always capture attention (Gibson, 1996a).

## General discussion

The present study describes three experiments testing the automatic nature of attention capture by multiple simultaneous abrupt onsets. Experiments 1 and 2 showed that when the onset information is irrelevant to the task, only one or two onsets capture attention automatically. Experiment 2 further showed that this is the case even when the onsets are readily distinguishable from the no-onset items. Experiment 3 showed that when the target type (i.e., onset or no-onset target) was known, participants were able to selectively search through all the onsets and up to four no-onset items.

These findings, especially in Experiment 1 and 2, are not in line with Yantis and Jones (1991) priority tag model. They proposed three possible models of capture, depending on whether none, one, or many onsets are prioritized. Models 2 and 3 where either one or multiple onsets respectively are automatically prioritized are particularly relevant to the present study According to Model 2, only one onset is prioritized during search and the RT to an onset target in a multiple onset display is mediated by the probability of the target being an onset. Such a model argues for the role of salience in attention capture and would include “abrupt onsets” in a category of features that capture attention. On the contrary, according to Model 3 all onsets are priority-tagged and searched before searching through the no-onset items. Priority tagging is assumed to be automatic and additional resources are required for their maintenance. This model emphasizes the special status of abrupt onsets in attention capture because onsets are the only features whose ability to capture attention extends beyond one object.

However, the findings in the present study prompts a re-evaluation of the role of higher-order mechanism like priority tags in attention capture by abrupt onsets. The three experiments presented here suggest that only one onset captures attention in a purely bottom-up automatic manner. The importance of this finding can be attributed to further confine and differentiate the role of onsets in attention capture. Overall, the finding suggests that a purely bottom up model of attention capture, which suggest that capture is triggered by an increased saliency signal, is sufficient to explain attention capture by abrupt onsets.

## Author note

This research was supported by a fellowship from the University of Warwick to Meera M. Sunny. Preliminary findings were presented at the Vision Science Society Meeting 2010 and 2011 in Naples, FL. We thank Dr. Derrick Watson for discussions and comments on an earlier draft of the manuscript and Dr. Ying Wang for her assistance with the modeling. Correspondence concerning this article should be addressed to Meera M. Sunny, Indian Institute of Technology-Gandhinagar, Ahmedabad, India. e-mail: m.m.sunny@iitgn.ac.in.

### Conflict of interest statement

The authors declare that the research was conducted in the absence of any commercial or financial relationships that could be construed as a potential conflict of interest.

## Footnotes

^1^The number of line segments that were deleted to form the no-onset letter was the same as the number of line segments that made up the onset letter.

^2^For a discussion of serial and parallel processing see Nakayama and Silverman (1986) and Townsend (1971, 1990)

^3^According to Yantis and Johnson (1990), all onsets are priority tagged, but search is possible only through a few of them as the tags decay over time. However, for simplicity we assume here that only a limited number of onsets is selected and that the estimated value will be equivalent to the number of onsets that can be searched before the tags fade away.

^4^The model also assumes that search efficiency (i.e., the time required to inspect one item) is constant. Search performance only varies as a function of inspection order, which is determined by the model parameters and the display factors.

^5^Because onset and no-onset targets were presented in separate blocks in two consecutive sessions the data was also analyzed including the additional factor Block Order. There was no effect involving Block Order, apart from its interaction with target type, *F* (1, 10) = 45.46, *p* < 0.05, which can easily be explained by the amount of practice participant had when they started the second block. Thus, participants who searched through onset items first did not have more difficulties in ignoring them in the consecutive session.
